# Accuracy improvement in protein complex prediction from protein interaction networks by refining cluster overlaps

**DOI:** 10.1186/1477-5956-10-S1-S3

**Published:** 2012-06-21

**Authors:** Tak Chien Chiam, Young-Rae Cho

**Affiliations:** 1 Department of Computer Science, Baylor University, Waco, Texas, USA; 2 Bioinformatics Program, Baylor University, Waco, Texas, USA

## Abstract

**Background:**

Recent computational techniques have facilitated analyzing genome-wide protein-protein interaction data for several model organisms. Various graph-clustering algorithms have been applied to protein interaction networks on the genomic scale for predicting the entire set of potential protein complexes. In particular, the density-based clustering algorithms which are able to generate overlapping clusters, i.e. the clusters sharing a set of nodes, are well-suited to protein complex detection because each protein could be a member of multiple complexes. However, their accuracy is still limited because of complex overlap patterns of their output clusters.

****Results**:**

We present a systematic approach of refining the overlapping clusters identified from protein interaction networks. We have designed novel metrics to assess cluster overlaps: overlap coverage and overlapping consistency. We then propose an overlap refinement algorithm. It takes as input the clusters produced by existing density-based graph-clustering methods and generates a set of refined clusters by parameterizing the metrics. To evaluate protein complex prediction accuracy, we used the *f*-measure by comparing each refined cluster to known protein complexes. The experimental results with the yeast protein-protein interaction data sets from BioGRID and DIP demonstrate that accuracy on protein complex prediction has increased significantly after refining cluster overlaps.

****Conclusions**:**

The effectiveness of the proposed cluster overlap refinement approach for protein complex detection has been validated in this study. Analyzing overlaps of the clusters from protein interaction networks is a crucial task for understanding of functional roles of proteins and topological characteristics of the functional systems.

## Background

Protein-protein interaction data are a crucial resource in understanding the underlying mechanisms of biological processes. In recent years, high-throughput experimental techniques have made remarkable advances in identifying protein-protein interactions on the scale of the entire genome, collectively referred to as the interactome. The rich amount of protein-protein interaction data sets have been integrated and mapped into a protein interaction network [1-3]. This network is represented as an undirected and un-weighted graph where proteins are nodes and interactions are edges.

Over the past few years, systematic analysis of protein interaction networks by theoretical and empirical studies has been in the spotlight in bioinformatics. It has been observed that the genome-scale interaction networks of several model organisms are typically modular [4]. Consequently, a wide range of graph clustering algorithms [5] have been applied to the interaction networks to predict potential protein complexes, the sets of proteins closely binding each other to perform specific cellular functions.

Previous graph clustering algorithms can be categorized into density-based approaches, hierarchical approaches and partition-based approaches. Density-based approaches detect densely connected subgraphs in protein interaction networks. A typical example in this category is the maximal clique algorithm to detect fully connected subgraphs [6]. Because of the strict constraints of the maximum-size cliques, relatively dense subgraphs are identified by using a density threshold or incorporating the percolation of small-size cliques. Because of computational inefficiency of finding cliques, a number of heuristic seed-growth style algorithms have been presented. They select seeds as initial points and expand them using alternative density functions. Typical examples include MCODE [7], DPClus [8], IPCA [9] and the entropy-based algorithm [10]. The details of these algorithms are discussed in the Method section.

The hierarchical approaches have been frequently applied to genomic or proteomic data because the hierarchical nature of clusters is significant to understand the global structure of functional organizations. Bottom-up hierarchical approaches start with each node as a separate cluster and then iteratively merge the two closest clusters. Top-down hierarchical approaches start with the whole graph as a single cluster and then recursively divide the cluster into smaller clusters. The iterative merging approaches should precisely measure distance or similarity between two clusters by estimating strength of interconnections or statistical significance of common members [11,12]. For the recursive division, finding exact cutting point for each iteration is a challenging issue. The edge-betweenness method [13] is an example to detect the hierarchy by identifying a bridge between two potential clusters repeatedly using the betweenness measure. The betweenness of an edge is calculated by the fraction of the shortest paths passing through the edge.

Partition-based approaches explore the best partition of a network, including the periphery. The Restricted Neighborhood Search Clustering (RNSC) [14] is a cost-based local search algorithm to find an optimal partition. The process begins with a random or user-specified partition. Each vertex on the border of a cluster is then moved to an adjacent cluster in a random manner such that cost is minimized. The cost function captures the ratio of invalid links between clusters to valid links within clusters. Markov Clustering (MCL) [15] is a fast and scalable partition-based algorithm by flow simulation. This algorithm simulates random walks within a Markov matrix that is mapped to the input graph. It repeatedly alternates between two operators, expansion and inflation, to update the matrix. This process continues until there is no further change in the matrix, terminating with the best partition of the graph.

Although these previous graph clustering algorithms are qualified to detect protein complexes from protein interaction networks, their accuracy is still limited. One of the challenges is overlapping cluster generation. The clustering algorithms should be able to assign each node to multiple clusters because a protein could have different interacting partners at different times and places. However, because the partition-based or hierarchical clustering algorithms always produce disjoint sets, only density-based methods are suitable for detecting overlapping clusters. A previous study [16] has presented a general model of overlapping sub-network structures. This model was validated by the intra-connection rate of each overlapping cluster.

We also note that the overlapping clusters sharing a set of proteins often represent the same protein complex. The examples in the protein interaction network of *S. cerevisiae *are shown in Table 1. For each cluster generated by the entropy-based approach, we computed *f*-measure by comparing to known protein complexes and chose the protein complex with the highest *f*-score. We observed in this test it occurs frequently that two or more clusters, in particular overlapping clusters, have the highest *f*-score to the same protein complex. In Table 1, the Prp19-associated complex is composed of eight proteins. The entropy-based method produced three overlapping clusters which have the best matches for the complex in *f*-measure. Four common proteins (YDR416W, YGR129W, YLL036C, YLR117C) over all three clusters are the members of the complex. The other four proteins in the complex also appear in one of the three clusters. How could we then infer a protein complex with higher accuracy from the overlapping clusters? The next three examples in Table 1 show the case that we can detect a protein complex with higher accuracy when we take the union set of two overlapping clusters. In contrast, the last example in table 1 is the case that the intersection set of two overlapping clusters matches a protein complex more precisely.

**Table 1:** Examples of overlapping clusters representing the same protein complex

Prp19-associated complex	
complex :	YLL036C YMR213W YJR050W YLR117C YDR416W YGR129W YBR188C YPR101W
cluster-1 :	YLL036C YDR416W YMR213W YGR129W YLR117C YNR011C YDR364C
cluster-2 :	YLL036C YJR050W YDR416W YGR129W YLR117C YPL213W YIR009W
cluster-3 :	YLL036C YDR416W YBR188C YGR129W YLR117C YPR101W
Set3p complex	
complex :	YGL194C YIL112W YDR155C YOL068C YKR029C YBR103W YCR033W
cluster-1:	YGL194C YKR029C YCR033W YIL112W
cluster-2 :	YGL194C YKR029C YBR103W

cAMP-dependent protein kinase	
complex :	YIL033C YJL164C YPL203W YKL166C
cluster-1 :	YNL227C YKL166C YPL203W
cluster-2 :	YIL033C YPL203W

NuA4 histone acetyltransferase complex	
complex :	YFL039C YJL081C YPR023C YEL018W YJR082C YNL136W YFL024C YOR244W YGR002C YHR099W YDR359C YNL107W YHR090C
cluster-1 :	YNL107W YOR244W YFL024C YPR023C
cluster-2 :	YJL081C YFL024C

RAVE complex	
complex :	YJR033C YDR202C YDR328C
cluster-1 :	YDR306C YDR202C YJL204C YGL149W YOR080W YJL149W YMR258C YBR280C YJR033C YML088W YDR131C YLR368W YLR097C YDL132W YLR352W YDR328C YLR224W
cluster-2 :	YMR054W YJR033C YDR202C YOR270C YBR127C YDL185W YHR060W

We applied the entropy-based approach to the yeast protein interaction network. For each cluster, we computed *f*-scores by comparing to experimentally determined protein complexes and chose one complex with the highest *f*-score. It shows five examples that two or more clusters have the highest *f*-score to the same protein complex.

In this article, we present a novel systematic approach to refine overlapping clusters and re-generate a new set of clusters from protein interaction networks. The aim of this study is to increase accuracy of protein complex prediction by refining the overlaps. First, we implement five density-based graph-clustering methods to obtain a set of preliminary overlapping clusters. We next introduce a unique strategy to refine the preliminary clusters by applying novel metrics: overlap coverage and overlapping consistency. We propose an overlap refinement algorithm which yields a final set of clusters by parameterizing the metrics. The experimental results with the protein-protein interaction data sets of *S. cerevisiae *downloaded from BioGRID [17] and DIP [18] show that the proposed approach achieves a statistically significant improvement on accuracy of protein complex prediction.

## Methods

### Previous density-based clustering methods

Density-based graph-clustering algorithms search densely connected subgraphs in protein interaction networks. We discuss four commonly-used methods in this category: CFinder, MCODE, DPClus and the entropy-based algorithm.

#### CFinder

Palla et al. [19] introduced a process of *k*-clique percolation along with the associated definitions of *k*-clique adjacency and *k*-clique chain. Two *k*-cliques are adjacent if they share (*k *− 1) nodes where *k *is the number of nodes in each clique. A *k*-clique chain is the union of a sequence of adjacent *k*-cliques. A *k*-clique percolation cluster is then a maximal *k*-clique chain. CFinder [20] searches all *k*-clique percolation clusters in an undirected graph with a parameter *k*. Larger *k *values correspond to a higher stringency during the identification of dense subgraphs and provide smaller groups with a higher density of links inside them.

#### MCODE

MCODE [7] is a typical seed-growth style clustering algorithm. It weights each node *v *by the core-clustering coefficient of *v*, which is defined as the density of the highest *k*-core of the directly connected neighbors of *v *together with *v *itself. Compared to the general clustering coefficient [21], the core clustering coefficient amplifies the weights of heavily interconnected regions while deleting many less-connected nodes. The *k*-core of a graph is a maximal subgraph such that each node in the subgraph has at least *k *links [22]. The algorithm then seeds a cluster with the highest weighted node and recursively includes a neighboring node if its weight is above a threshold.

#### DPClus

DPClus [8] is also a seed-growth algorithm to find local dense regions based on connectivity. It weights each node by sum of the edge weights to its neighboring nodes, while each edge is weighted by the number of common neighbors between two ending nodes. The node with the highest weight is selected as a seed which becomes a single-node cluster. The cluster grows gradually by adding repeatedly its neighboring nodes if it reaches a density threshold for either the core or the periphery. IPCA [9] has the same process to DPClus on weighting nodes and selecting a seed. However, on the step of extending the seed cluster, a neighboring node is added if it has a higher ratio of links to the cluster than an interaction probability threshold and if the diameter of the cluster is less than a maximum diameter threshold.

#### **Entropy-based algorithm**

The entropy-based approach [10] has been recently introduced as a seed-growth algorithm. It repeatedly finds a locally optimal cluster with minimal graph entropy. A high-level description of the algorithm is given below:

1. Select a random seed node, and form a seed cluster including the selected seed and its neighbors.

2. Remove nodes in the cluster iteratively to decrease graph entropy until it is minimal.

3. Add neighboring nodes of the cluster iteratively to decrease graph entropy until it is minimal.

4. Output the cluster, and repeat the steps (1), (2) and (3) until no seed candidate remain.

As a weakness, this algorithm might fall into the local minimum too quickly. To avoid this problem, we propose a slight variation of the entropy-based clustering algorithm. (It will be called the modified entropy-based method.) A high-level description of the algorithm is given below:

1. Select a clique of size 3 as an initial cluster.

2. Add all neighboring nodes of the cluster.

3. Remove nodes added on the step (2) iteratively to decrease graph entropy until it is minimal.

4. Repeat the steps (2) and (3) until the step (3) removes all nodes added on the step (2).

5. Output the cluster, and repeat the steps from (1) to (4) until no seed candidate remain.

This modification allows the clusters to keep growing in the case where the addition of a neighboring node will temporarily increase entropy, but the addition of that node along with certain additional neighboring nodes will ultimately decrease entropy. For example, if there exists a set of densely connected neighboring nodes of a cluster, the original algorithm will only consider each node independently. However, the modified algorithm will consider the set as a whole.

### Cluster overlap analysis

In this section, we introduce novel metrics to define properties of cluster overlaps. Suppose we have a set of *n *clusters. An overlap is a non-empty intersection of two clusters. Then, the overlaps of a cluster *c_i _*can be defined as a non-unique collection of sets of vertices in *c_i_*, each of which is an overlap of *c_i _*with another cluster. When *V *(*c_i_*) denotes the set of all vertices in *c_i_*,

(1) $$Overlaps\left(c_{i}\right)=\left\{V\left(c_{i}\right)\cap V\left(c_{j}\right)|1\leq j\leq n,j\neq i\right\},$$

where *V *(*c_i_*) ∩ *V *(*c_j_*) ≠ ∅. The cluster *c_i _*may have overlaps with several other clusters, and each overlap may have the different number of vertices. The average overlap size of a cluster *c_i _*is then formulated as

(2) $$S_{overlap}\left(c_{i}\right)=\frac{\sum_{o\in Overlaps\left(c_{i}\right)}\left|o\right|}{\left|Overlaps\left(c_{i}\right)\right|}$$

where *|o*| is the size of the overlap *o*.

#### Overlap rate

The overlap rate of a cluster *c_i _*is defined as the average overlap size of *c_i_*, divided by the total number of vertices in *c_i_*.

(3) $$R_{overlap}\left(c_{i}\right)=\frac{S_{overlap}\left(c_{i}\right)}{\left|V\left(c_{i}\right)\right|}$$

This formula indicates the fraction of the vertices in *c_i _*involved in the average overlap. Higher the overlap rate of *c_i _*is, more vertices in *c_i _*appear in any other clusters on average.

#### Overlap coverage

The overlap coverage of a cluster *c_i _*represents the ratio of the number of vertices in *c_i _*which appear in one or more overlaps of *c_i_*.

(4) $$Cov\left(c_{i}\right)=\frac{\left|{⋃}_{o\in Overlaps\left(c_{i}\right)}o\right|}{\left|V\left(c_{i}\right)\right|}$$

This formula can be used to measure how unique the cluster *c_i _*is. Higher overlap coverage of *c_i _*indicates that a larger portion of the vertices in *ci *are also included into other clusters. For instance, if all vertices in *c_i _*are shared with other clusters, then *c_i _*has the maximum overlap coverage which is 1.

#### Overlapping consistency

The overlapping consistency of a cluster *c_i _*measures the uniformity of the overlaps of *c_i_*. It is calculated as the overlap rate divided by the overlap coverage.

(5) $$Cons\left(c_{i}\right)=\frac{R_{overlap}\left(c_{i}\right)}{Cov\left(c_{i}\right)}$$

The overlapping consistency ranges between 0 and 1, inclusive, because the values for *R_overlap_*(*c_i_*) are upper-bounded by the values of *Cov*(*c_i_*). For instance, if a vertex in *c_i _*also belongs to several different clusters and the other vertices in *c_i _*do not belong to any other clusters, then *c_i _*has the maximum overlapping consistency because the overlap rate and overlap coverage are the same. If both of the overlapping consistency and the overlap coverage are high, this could indicate the overlapping clusters represent highly related groups.

### Cluster overlap refinement

We propose an algorithm for refining the preliminary overlapping clusters using the novel metrics defined above. This method creates a new cluster from the preliminary clusters that have significant overlaps by parameterizing the metrics. This cluster overlap refinement algorithm is described in Table 2.

**Table 2:** The cluster overlap refinement algorithm

OverlapOptimization (*S, minCov, minCons, minCss*)
1 **for each ***g *∈ *S*
2 **if ***Cov*(*g*) <*minCov *or *Cons*(*g*) <*minCons*
3 Add *g *into *S*'
4 **end if**
5 **else**
6 Assign all nodes a value of 0
7 Increment the value of each node in *g*
8 *count *← 1
9 Find overlapping clusters with *g*
10 **for each **overlapping cluster *c*
11 *g *← *g *∪ *c*
12 Increment the value of each node in *c*
13 *count *← *count *+1
14 **end for**
15 Remove from *g *any node with a value less than (*n *× *minCss*)
16 **if ***g *is not redundant
17 Add *g *into *S*'
18 **end if**
19 **end else**
20 **end for**
21 **return *S*'**
__________________________________________________________________________

This algorithm takes as input a set of preliminary clusters, *S*, generated by any density-based clustering methods. It returns the refined set of clusters, *S*', as output.

The algorithm takes as input a set of preliminary clusters, *S*. It requires three parameters as thresholds: the minimum overlap coverage *minCov*, the minimum overlapping consistency *minCons*, and the minimum consensus constraint *minCss*. In Line 2 of the algorithm, the *minCov *and *minCons *become the minimum boundaries of overlap coverage and consistency for each cluster to be refined. Line 15 enforces the consensus constraint to merge clusters only if they are strongly related. This constraint changes the overlap optimizing process. If this minimum consensus constraint *minCss *was 100%, then the result would be the intersection of the overlapping clusters. If it was 0%, the result would be the union of them. This constraint can thus be chosen flexibly between the intersection and the union to select only significant vertices from overlapping clusters. The proper selection of the minimum consensus value prevents a set of clusters from being generated by the two extreme cases of the union, which is too generous, and the intersection, which is too strict.

### Clustering accuracy measure

For clustering accuracy evaluation, we compare each cluster to real protein complexes using the *f*-measure as a combination of precision and recall. Suppose we compare a cluster *c *with a protein complex *p_i_*. Recall, also called a true positive rate or sensitivity, is the ratio of common members of *c *and *p_i _*to the number of proteins in *p_i_*.

(6) $$Recall=\frac{\left|c\cap p_{i}\right|}{\left|p_{i}\right|}$$

Precision, also called a positive predictive value, is the ratio of common proteins of *c *and *p_i _*to the number of proteins in *c*.

(7) $$Precision=\frac{\left|c\cap p_{i}\right|}{\left|c\right|}$$

The *f*-score is then the harmonic mean of recall and precision.

(8) $$f=\frac{2\times Recall\times Precision}{Recall+Precision}$$

This *f*-score makes a direct comparison between an output cluster and a gold-standard protein complex without any bias towards the cluster size. For each output cluster, we search for the best match from the list of gold-standard protein complexes in regard to *f*-scores. The accuracy of clustering algorithms is then measured by the average *f*-score of the best matches over all output clusters.

## Results and discussion

### Data source

We explored the application of our approach to protein-protein interaction data of *S. cerevisiae*. The genome-wide yeast protein-protein interaction data are publicly available in several open databases such as BioGRID [17], IntAct [23], MINT [24], MIPS [25], STRING [26] and DIP [18]. In this experiment, we used two protein-protein interaction data sets. First, we downloaded the core protein-protein interaction data of *S. cerevisiae *from DIP, which includes 2526 distinct proteins and 5949 interactions between them. The core interactions have been selected from the full data set by curative processes based on protein sequences and RNA expression profiles [27]. We thus expect that most of the interactions in this data set are reliable. However, we have to consider a number of false negatives, i.e. true interactions which do not appear in this data set. Next, we tested with the exceptionally large protein-protein interaction data set of *S. cerevisiae *from BioGRID, which includes 5590 distinct proteins and 92906 interactions. This data set has been accumulated from high-throughput experimental results published. It is therefore likely to contain a significant number of false positives, i.e. spurious interactions which do not occur in vivo.

To evaluate clustering accuracy of the proposed approach, we acquired the protein complex data recently determined [28]. As gold-standard, we combined both data sets: CYC2008 which has 408 manually curated heteromeric protein complexes derived from small-scale experiments and YHTP2008 which comprises 400 putative complexes collected mostly from high-throughput experimental results.

### Protein complex detection from DIP data

#### Clustering by existing methods

**Figure 1:**
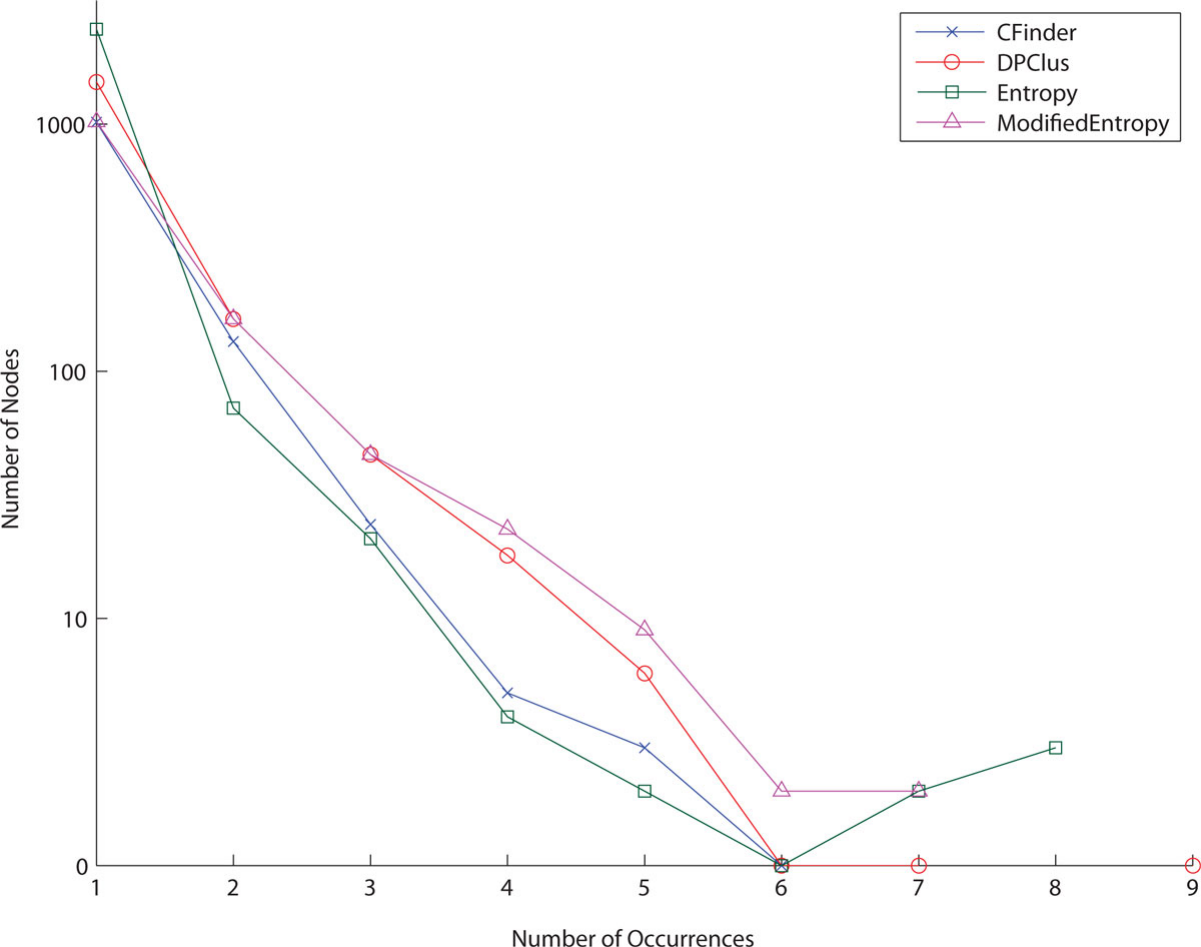
**Distribution of occurrences of any protein over all clusters**. We counted how many times each protein occurs in different clusters. Each plot describes the exponential decrease of the number of proteins with respect to the number of occurrences. All four clustering algorithms have a similar trend of protein occurrences in overlapping clusters.

To predict potential protein complexes from DIP protein-protein interaction data, we tested five density-based graph clustering approaches: CFinder, MCODE, DPClus, the entropy-based method, and the modified entropy-based method. Their clustering results are shown in Table 3. The entropy-based method produced a large number of small-sized clusters including many singletons, i.e. clusters containing only a single protein, whereas the modified entropy-based method generated a small number of large-sized clusters. The output clusters of CFinder has the highest average overlap rate which is close to 0.2. The overlap rate of a cluster indicates the proportion of overlaps in the cluster on average. The average overlap rate of 0.2 thus implies that 20% of the nodes in each cluster are involved in overlapping on average. Interestingly, MCODE was not able to yield any overlapping clusters although it searches densely connected sub-graphs. For each method, the distribution of occurrences of any protein over all output clusters is plotted in a log scale in Figure 1. We counted how many times each protein occurs in different clusters. As a general pattern of output clusters, these plots describe the exponential decrease of the number of proteins with respect to the number of occurrences. In this experiment, a slightly different trend from the average overlap rate on CFinder was perceptible. CFinder generated less proteins occurring in multiple clusters than DPClus and the modified entropy-based method. This result implies that CFinder produces the clusters with higher overlapping consistency because of a higher overlap rate, but lower overlap coverage because of less proteins involved in overlapping, than the other methods.

**Table 3:** Clustering results of five density-based approaches and their accuracy on DIP data

method	number of clusters	average overlap rate	average *f*-score
CFinder	172	0.199	0.602
MCODE	272	0.000	0.456
DPClus	449	0.160	0.473
Entropy	1294	0.060	0.309
Modified-Entropy	110	0.099	0.485

We tested five density-based graph-clustering approaches on the yeast protein-protein interaction data from DIP. Their clustering results and the average overlap rates are shown. The overlap rate of a cluster indicates the proportion of overlaps in the cluster on average. As accuracy of each method on predicting protein complexes, we measured the average *f*-score of the clusters comparing to protein complexes experimentally determined.

To evaluate accuracy of each method, we measured the average *f*-score of output clusters comparing to gold-standard protein complexes. As shown in Table 3, the clusters generated by CFinder have the highest average *f*-score. However, as a drawback, CFinder requires the longest runtime in the large-size complex network among all the methods tested. The clusters generated by the entropy-based method have the lowest average *f*-score because most of them are extremely small-sized. However, the modification of this method has markedly improved its accuracy by yielding relatively large clusters, and achieved a slightly higher level of accuracy than MCODE and DPClus.

#### Improvement by cluster overlap refinement

We implemented the cluster overlap refinement approach to assess improvement on protein complex detection. We used as input the set of clusters produced by three clustering algorithms: CFinder, DPClus and the modified entropy-based method. We were not able to test MCODE because the clusters did not have any overlaps. We also dropped testing the original entropy-based method because the average overlapping rate is close to 0. Instead of the entropy-based method, we used the modified entropy-based method for this experiment. The optimal refinement of cluster overlaps was performed by changing the values of three parameters: the minimum overlap coverage threshold (minCov), the minimum overlapping consistency threshold (minCons) and the minimum consensus constraint (minCss). It collected all overlapping clusters which have the overlap coverage and the overlapping consistency greater than their minimum thresholds, and then re-generated a new set of clusters by selecting the optimal value of minCss.

**Figure 2:**
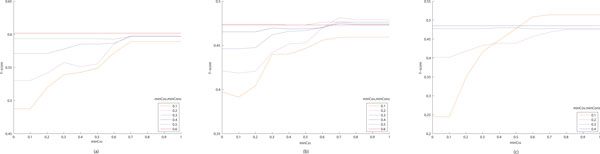
**Accuracy change with different parameter settings for minCss on refining cluster overlaps from DIP data**. These figures show how the average *f*-score of the clusters refined is affected by different parameter values of minCss. We used as input the preliminary clusters produced by (a) CFinder, (b) DPClus and (c) the modified entropy-based method. We varied the parameter values of minCov and minCons from 0.1 to 0.6. At low values of minCov and minCons, we have a low average *f*-score when minCss is lower than 0.3. As minCov and minCons increase, we have consistent average *f*-scores regardless of minCss values.

First, we used varying parameter values of minCov and minCons between 0 and 1 to explore the effect of minCss on protein complex detection accuracy. Since the minimum consensus of 0 means taking the union of all overlapping clusters, the output clusters are gradually enlarged as minCss decreases. In contrast, the minimum consensus of 1, as taking the intersection of overlapping clusters, resulted in the lowest average cluster size. Figure 2 shows how accuracy of the clusters refined is affected by different values of minCss. Figure 2(a), (b) and 2(c) are the results when we used the preliminary clusters produced by CFinder, DPClus and the modified entropy-based method, respectively. We varied the parameter values of minCov and minCons from 0.1 to 0.6, but assigned the same value to minCov and minCons for each case. Use of low values of minCov and minCons means that the overlap refinement process is applied to the clusters even if they have only a small portion of overlaps. Therefore, when minCov and minCons are 0.1 or 0.2, we could observe a pattern such that the average *f*-score is very low when minCss is lower than 0.3. It is readily understood that naively merging two clusters results in low accuracy. As minCov and minCons increase, we have consistent average *f*-scores regardless of minCss values. If two clusters have a very large overlapping region, then their union set would be similar to their overlap. In the tests of CFinder and DPClus, the average *f*-scores were not affected by changing minCss when minCov and minCons are 0.6. For the modified entropy-based method, we attained the consistent average *f*-score when assigning 0.4 to both minCov and minCons. Considering all plots in Figure 2, we chose as the optimal value of minCss 0.7 for CFinder and DPClus and 0.8 for the modified entropy-based method.

**Figure 3:**
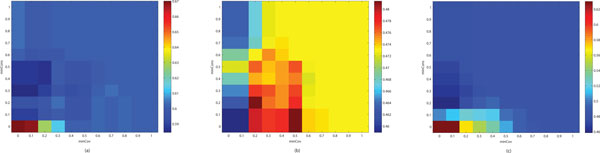
**Accuracy change with different parameter settings for minCov and minCons on refining cluster overlaps from DIP data**. These figures show the effect of different parameter values for minCov and minCons on the average *f*-score of the clusters refined. We used as input the preliminary clusters produced by (a) CFinder, (b) DPClus and (c) the modified entropy-based method. For minCss, we used the optimal values selected from the experimental results in Figure 2, 0.7 in (a) and (b) and 0.8 in (c). In (a) and (c), the best average *f*-scores of the refined clusters are achieved with the lowest values of minCov and minCons. In (b), the best accuracy is captured in the ranges between 0.2 and 0.5 for minCov and between 0 and 0.2 for minCons.

Next, we used the selected minCss values to find the optimal combination of minCov and minCons. Figure 3 shows the effect of different parameter settings for minCov and minCons on the accuracy of the clusters refined. Figure 3(a), (b) and 3(c) show the results from CFinder, DPClus and the modified entropy-based method, respectively. In the tests of CFinder and the modified entropy-based method, we achieved the best average *f*-scores of refined clusters when using the lowest values of minCov and minCons. This trend was already observed in the previous experiment for the modified entropy-based method in Figure 2(c). However, for DPClus, the best accuracy was captured in the ranges between 0.2 and 0.5 for minCov and between 0 and 0.2 for minCons, as shown in Figure 3(c).

**Figure 4:**
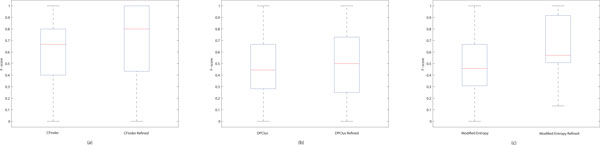
**Statistical analysis of accuracy improvement on protein complex detection by refining cluster overlaps from DIP data**. For each method, two distributions of *f*-scores of the clusters before and after refining overlaps are exhibited. The box plots in (a) and (c) show a substantial increase of the 1st quartile, the median and the 3rd quartile points after refining overlaps. The box plots in (b) also show a slight increment of the median and the 3rd quartile points after refining overlaps. These figures justify the effectiveness of the proposed overlap refinement approach.

We analyzed statistically the improvement on protein complex detection by refining cluster overlaps. Figure 4 displays in box plots two distributions of *f*-scores of the clusters before and after overlap refinement for each of the three clustering algorithms. Figure 4(a) obviously demonstrates that the overall accuracy of the clusters produced by CFinder has been improved by refining overlaps because of more than 15% increase of the median point and more than 20% increase of the 3rd quartile (the upper quartile) point in the *f*-score distribution. Because the clusters which have the overlap coverage and overlapping consistency below the selected thresholds remain intact during the refinement, it is feasible that the 1st quartile (the lower quartile) or the minimum point does not alter in the distribution. As shown in Figure 4(b), the accuracy of the clusters produced by DPClus has been slightly improved by refining overlaps. However, the refinement approach has improved substantially the clusters produced by the modified entropy-based method. Figure 4(c) shows the increments of both the 1st and 3rd quartile points. These results in Figure 4 justify the effectiveness of the proposed overlap refinement approach. They also address that the extent of improvements varies depending on the clustering algorithms and their preliminary clusters.

### Protein complex detection from BioGRID data

We carried out additional experiments of cluster overlap refinement with the most recent version of the protein-protein interaction data set of *S. cerevisiae *from BioGRID. This BioGRID interaction network is larger and significantly denser than the DIP network, 2.2 times more distinct proteins and 15 times more edges. Moreover, it has been considered that it includes a large number of false interactions which create extremely complex connectivity. It is thus expected that the accuracy of protein complex detection from BioGRID data is lower than the previous tests with DIP data.

We tested five density-based graph clustering approaches: CFinder, MCODE, DPClus, the entropy-based method, and the modified entropy-based method. However, we were not able to obtain clustering results from CFinder because of its computational inefficiency on the large and dense network. Same to the previous test, MCODE did not generate any overlapping clusters. The entropy-based method produced an extremely large number of singletons and very few clusters containing more than 3 proteins. These clusters are also non-overlapping. Table 4 shows the clustering results and their accuracy. As compared to Table 3, all the methods, except the entropy-based method, generated more clusters with similar overlapping rates. However, as expected, the average *f*-scores of the clusters decreased remarkably on this complex network.

**Table 4:** Clustering results of four density-based approaches and their accuracy on BioGRID data

method	number of clusters	average overlap rate	average *f*-score
MCODE	301	0.000	0.229
DPClus	696	0.167	0.331
Entropy	47	0.000	0.169
Modified-Entropy	243	0.094	0.175

We tested four density-based graph-clustering approaches on the yeast protein-protein interaction data from BioGRID. Their clustering results and the average overlap rates are shown. As accuracy of each method on predicting protein complexes, we measured the average *f*-score of the clusters comparing to protein complexes experimentally determined.

**Figure 5:**
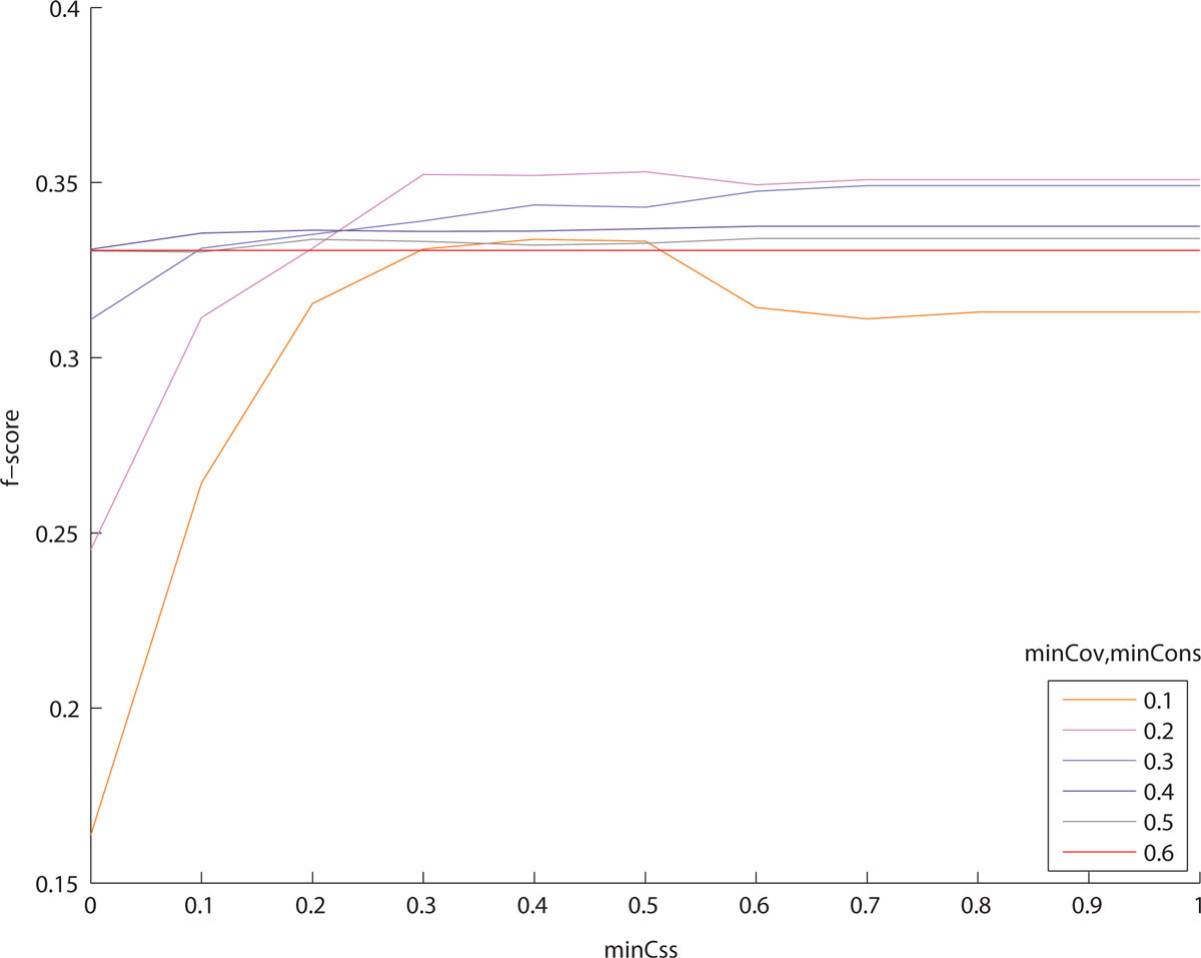
**Accuracy change with different parameter settings for minCss on refining cluster overlaps from BioGRID data**. This figure shows how the average *f*-score of the clusters refined is affected by different parameter values of minCss. We used as input the preliminary clusters produced by DPClus. At low values of minCov and minCons, we have a low average *f*-score when minCss is lower than 0.2. As minCov and minCons increase, we have consistent average *f*-scores regardless of minCss values.

**Figure 6:**
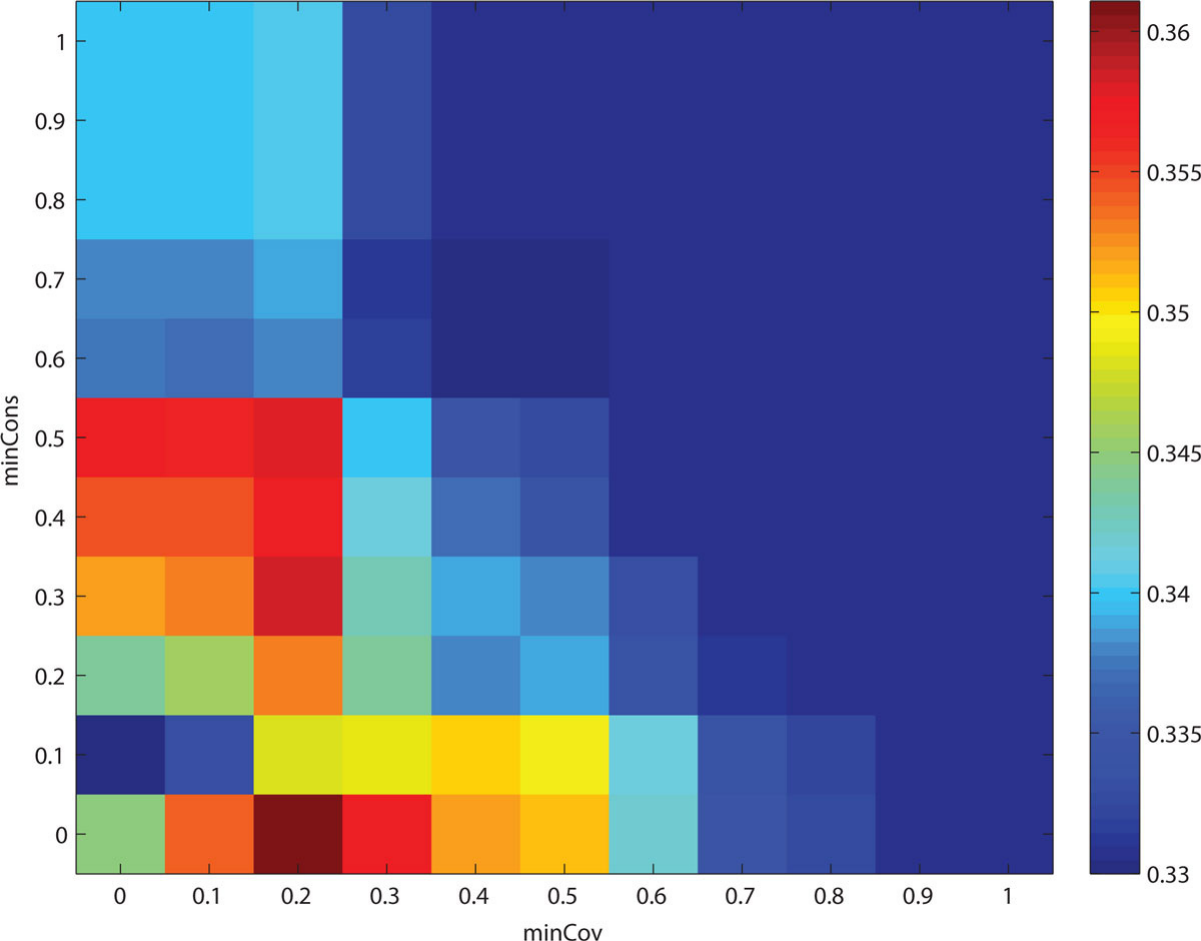
**Accuracy change with different parameter settings for minCov and minCons on refining cluster overlaps from BioGRID data**. This figure shows the effect of different parameter values for minCov and minCons on the average *f*-score of the clusters refined. We used as input the preliminary clusters produced by DPClus. We used minCss of 0.4. The parameter values, minCov of 0.2 and minCons of 0, can be chosen as the best combination.

**Figure 7:**
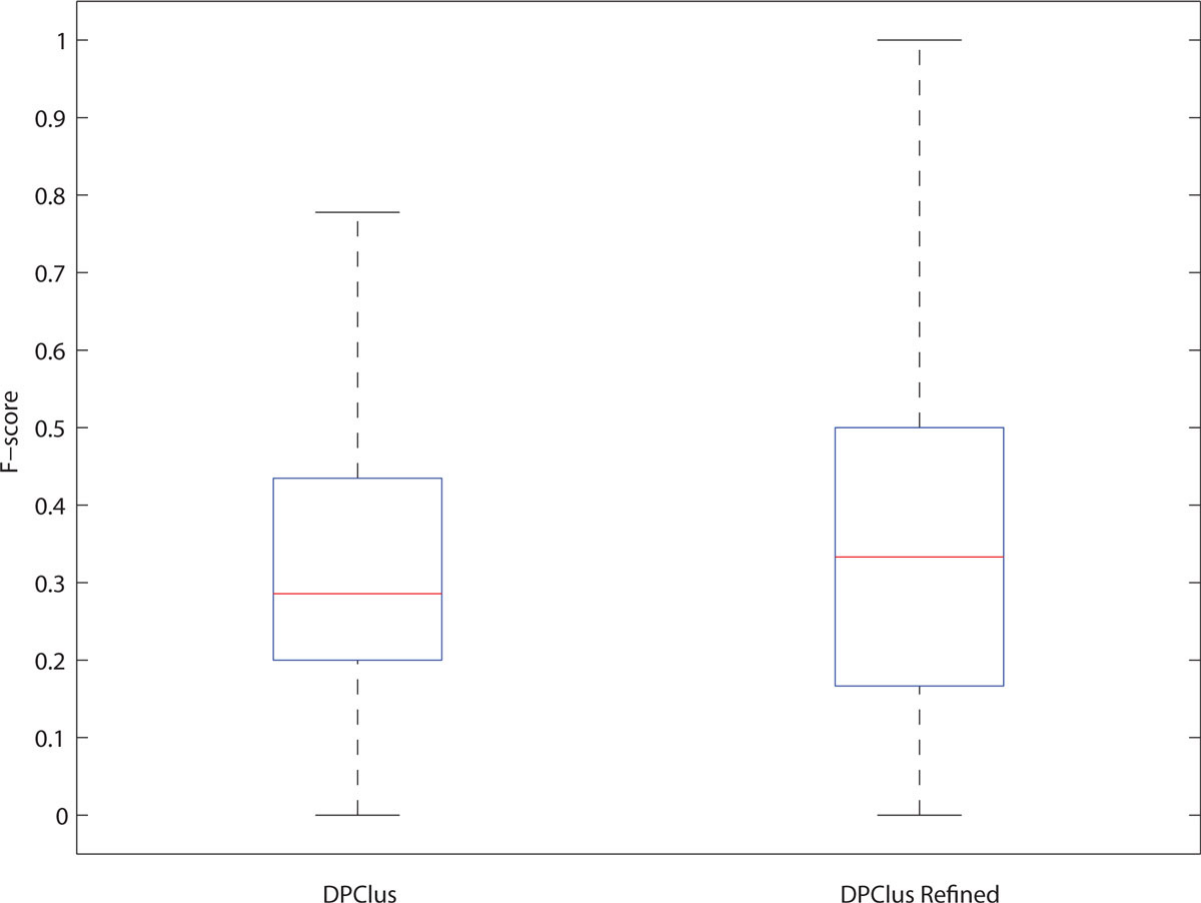
**Statistical analysis of accuracy improvement on protein complex detection by refining cluster overlaps from BioGRID data**. Two distributions of *f*-scores of the clusters produced by DPClus before and after refining overlaps are shown. The 3rd quartile and the maximum points significantly increased after refining overlaps.

To select the optimal parameter values of minCov, minCons and minCss for this data set, we applied the same procedure as discussed in the previous section. We dropped testing MCODE and the entropy-based method because of the overlapping rate of 0. We also failed testing the modified entropy-based method because it generated the clusters with lower overlapping consistency, which can be verified by its lower average overlap rate, but extremely higher overlap coverage than the other methods. As a result, the overlap refinement process terminated with merging all output clusters into a single cluster. We thus used the set of clusters produced by DPClus only. First, we changed the values of minCov and minCons to find the best minCss, as shown in Figure 5. The general changing pattern of the average *f*-score was similar to that in Figure 2(b). As minCov and minCons increase, we have consistent average *f*-scores regardless of minCss values. At low values of minCov and minCons, the average *f*-score is very low when minCss is lower than 0.1. This plot shows that the optimal minCss value should be selected in the range between 0.4 and 0.5, which is lower than the optimal value chosen in the previous section. We next used the minCss value of 0.4 to find the optimal combination of minCov and minCons. Figure 6 shows the average *f*-score change by different parameter settings of minCov and minCons. From this result, minCov of 0.2 and minCons of 0 should be chosen as the best combination. Figure 7 shows statistical analysis for the improvement on protein complex detection by overlap refinement. The 3rd quartile (the upper quartile) and the maximum points significantly increased after refining overlaps. This result also indicates that the proposed overlap refinement approach works effectively on large-size complex networks.

## Conclusion

The generation of the genome-wide protein-protein interactions in model organisms is proceeding rapidly, heightening the demand for advances in the computational techniques to provide systematic mapping and analyze the protein interaction networks. Advanced computational approaches have been applied to uncover functional patterns hidden in the complex systems. In particular, various graph-clustering algorithms have identified potential functional organizations from protein interaction networks.

We have designed a novel approach of analyzing cluster overlaps systematically. Our approach refines the overlapping clusters, generated by any commonly-used density-based clustering techniques, for the purpose of increasing accuracy on protein complex prediction from protein interaction networks. Through a series of newly defined overlap formulas such as overlap coverage and overlapping consistency, the proposed overlap refinement algorithm enhances the quality of the clusters best matching to known protein complexes.

The proposed approach has been tested with two yeast protein-protein interaction data sets: BioGRID which is known as complete interactome and the core set from DIP which is a reliable subset of full data. The preliminary clusters as input have been acquired from several density-based clustering algorithms: CFinder, MCODE, DPClus and the entropy-based method. We discussed the process of finding the best parameter settings for minCov, minCons and minCss in the proposed approach. We finally demonstrated significant improvements on protein complex prediction accuracy after refining preliminary overlapping clusters. These experimental results eventually led to the conclusion that this approach works successfully for any clustering methods and any protein-protein interaction data sets by optimizing the parameter values.

Overlapping is one of the key properties of functional organizations of molecular components. Analyzing the overlaps of clusters from protein interaction networks is a critical task for not only detecting protein complexes but also complete understanding of functional roles of proteins and topological characteristics of the functional systems. This study provides a systematic framework for effective analysis of functional overlap information inherent in biological networks.

## Competing interests

The authors declare that they have no competing interests.

## Authors' contributions

TCC implemented the proposed approach and tested its performance. YRC designed the experimental process, analyzed the results and wrote the manuscript. They both read and approved the final manuscript.

## References

[B1] Rual J-F Towards a proteome-scale map of the human protein-protein interaction network Nature 2005 437 1173 1178 10.1038/nature04209 16189514

[B2] Yu H High-quality binary protein interaction map of the yeast interactome network Science 2008 322 104 110 10.1126/science.1158684 18719252 PMC2746753

[B3] Venkatesan K An empirical framework for binary interactome mapping Nature Method 2009 6 1 83 90 10.1038/nmeth.1280 PMC2872561 19060904

[B4] Barabasi A-L Oltvai ZN Network biology: understanding the cell's functional organization Nature Reviews: Genetics 2004 5 101 113 10.1038/nrg1272 14735121

[B5] Li X Wu M Kwoh C-K Ng S-K Computational approaches for detecting protein complexes from protein interaction networks: a survey BMC Genomics 2010 11 Suppl 1 S3 10.1186/1471-2164-11-S1-S3 20158874 PMC2822531

[B6] Spirin V Mirny LA Protein complexes and functional modules in molecular networks Proc Natl Acad Sci USA 2003 100 21 12123 12128 10.1073/pnas.2032324100 14517352 PMC218723

[B7] Bader GD Hogue CW An automated method for finding molecular complexes in large protein interaction networks BMC Bioinformatics 2003 4 2 10.1186/1471-2105-4-2 12525261 PMC149346

[B8] Altaf-Ul-Amin M Shinbo Y Mihara K Kurokawa K Kanaya S Development and implementation of an algorithm for detection of protein complexes in large interaction networks BMC Bioinformatics 2006 7 207 10.1186/1471-2105-7-207 16613608 PMC1473204

[B9] Li M Chen J Wang J Hu B Chen G Modifying the DPClus algorithm for identifying protein complexes based on new topological structures BMC Bioinformatics 2008 9 398 10.1186/1471-2105-9-398 18816408 PMC2570695

[B10] Kenley EC Cho Y-R Detecting protein complexes and functional modules from protein interaction networks: A graph entropy approach Proteomics 2011 11 19 3835 3844 10.1002/pmic.201100193

[B11] Brun C Herrmann C Guenoche A Clustering proteins from interaction networks for the prediction of cellular functions BMC Bioinformatics 2004 5 95 10.1186/1471-2105-5-95 15251039 PMC487898

[B12] Samanta MP Liang S Predicting protein functions from redundancies in large-scale protein interaction networks Proc Natl Acad Sci USA 2003 100 22 12579 12583 10.1073/pnas.2132527100 14566057 PMC240660

[B13] Dunn R Dudbridge F Sanderson CM The use of edge-betweenness clustering to investigate biological function in protein interaction networks BMC Bioinformatics 2005 6 39 10.1186/1471-2105-6-39 15740614 PMC555937

[B14] King AD Przulj N Jurisica I Protein complex prediction via cost-based clustering Bioinformatics 2004 20 17 3013 3020 10.1093/bioinformatics/bth351 15180928

[B15] Van Dongen S A new clustering algorithm for graphs 2000 Tech Rep INS-R0010, National Research Institute for Mathematics and Computer Science in the Netherlands

[B16] Cho Y-R Hwang W Ramanathan M Zhang A Semantic integration to identify overlapping functional modules in protein interaction networks BMC Bioinformatics 2007 8 265 10.1186/1471-2105-8-265 17650343 PMC1971074

[B17] Stark C The BioGRID interaction database: 2011 update Nucleic Acids Research 2011 39 D698 D704 10.1093/nar/gkq1116 21071413 PMC3013707

[B18] Salwinski L Miller CS Smith AJ Pettit FK Bowie JU Eisenberg D The database of interacting proteins: 2004 update Nucleic Acids Research 2004 32 D449 D451 10.1093/nar/gkh086 14681454 PMC308820

[B19] Palla G Derenyi I Farkas I Vicsek T Uncovering the overlapping community structure of complex networks in nature and society Nature 2005 435 814 818 10.1038/nature03607 15944704

[B20] Adamcsek B Palla G Farkas IJ Derenyi I Vicsek T CFinder: locating cliques and overlapping modules in biological networks Bioinformatics 2006 22 8 1021 1023 10.1093/bioinformatics/btl039 16473872

[B21] Watts DJ Strogatz SH Collective dynamics of 'small-world' networks Nature 1998 393 440 442 10.1038/30918 9623998

[B22] Wuchty S Almaas E Peeling the yeast protein network Proteomics 2005 5 444 449 10.1002/pmic.200400962 15627958

[B23] Aranda B The IntAct molecular interaction database in 2010 Nucleic Acids Research 2010 38 D525 D531 10.1093/nar/gkp878 19850723 PMC2808934

[B24] Ceol A Chatr-aryamontri A Licata L Peluso D Briganti L Perfetto L Castagnoli L Cesareni G MINT: the molecular interaction database: 2009 update Nucleic Acids Research 2010 38 D532 D539 10.1093/nar/gkp983 19897547 PMC2808973

[B25] Mewes HW MIPS: analysis and annotation of genome information in 2007 Nucleic Acids Research 2008 36 D196 D201 18158298 10.1093/nar/gkm980 PMC2238900

[B26] von Mering C Jensen LJ Kuhn M Chaffron S Doerks T Kruger B Snel B Bork P STRING7-recent developments in the integration and prediction of protein interactions Nucleic Acids Research 2007 35 D358 D362 10.1093/nar/gkl825 17098935 PMC1669762

[B27] Deane CM Salwinski L Xenarios I Eisenberg D Protein interactions: two methods for assessment of the reliability of high throughput observations Molecular and Cellular Proteomics 2002 1 349 356 10.1074/mcp.M100037-MCP200 12118076

[B28] Pu S Wong J Turner B Cho E Wodak SJ Up-to-date catalogues of yeast protein complexes Nucleic Acids Research 2009 37 3 825 831 10.1093/nar/gkn1005 19095691 PMC2647312

